# The Gut-Brain Connection: Do Gut Bacteria Play a Role in the Development of Dementia? A Systematic Review

**DOI:** 10.1192/bjo.2024.226

**Published:** 2024-08-01

**Authors:** Prakriti Pokharel

**Affiliations:** Surrey and Borders NHS Foundation Trust, Surrey, United Kingdom. Brighton and Sussex Medical School, Brighton, United Kingdom

## Abstract

**Aims:**

Dementia is a debilitating disease with multiple potential causes, no cure and rising incidence. New studies suggest that gut bacteria not only aid in the digestion of bowel products but also other bodily functions such as immune systems and relaying messages to the brain. This review aims to examine the potential link between gut microbiota and dementia by performing a systematic review to assess whether gut bacteria play a role in the development of dementia.

**Methods:**

The systematic review was designed and conducted according to PRISMA guidelines. A modified PICO model was used to perform a literature search in Medline, CINAHL PLUS and APA PsychInfo databases. The search identified 401 articles, 49 of which met the predefined inclusion criteria. Twenty-one final studies were included in the results; 14 cross-sectional, two cohort, three case-control, one randomised control study and one case report. The reviewer extracted and analysed data from these studies for quality using the AXIS and CASP tools. A narrative synthesis of the results was performed due to the heterogeneity of the data.

**Results:**

Individuals with dementia have lower microbial diversity than healthy controls, including changes in specific bacterial taxa, pro-inflammatory and anti-inflammatory balance. The results of the review were subdivided into four identified themes which helped further identify that microbial metabolites, diet and gastrointestinal disease can also influence the composition of gut microbiomes and, therefore, the development of cognitive impairment and dementia.

**Conclusion:**

This systematic review found a link between gut bacteria, bacterial metabolites, gastrointestinal health, diet, and dementia. Although the studies were mostly observational, they suggest that gut microbiota can affect brain function through dysbiosis, which can lead to neuroinflammation and dementia. More research is needed to confirm a causal relationship, but targeting the gut microbiota could be a potential therapy for MCI and AD. Innovative strategies may help combat the growing challenge of dementia.

